# Divergent Effects of Peripheral vs. Central Oxytocin Administration on Observational Fear Behavior in Male and Female Mice

**DOI:** 10.3390/ph19030350

**Published:** 2026-02-24

**Authors:** Yuan Fu, Shufang Feng, Wenlong Shi, Yu Qin, Tianyao Shi, Wenxia Zhou

**Affiliations:** 1 State Key Laboratory of Toxicology and Medical Countermeasures, Beijing Institute of Pharmacology and Toxicology, Beijing 100850, China; 2 Department of Pharmacy, Nanjing University of Chinese Medicine, Nanjing 210023, China; 3 Department of Gerontology, The Third Medical Center, Chinese PLA General Hospital, Beijing 100039, China

**Keywords:** oxytocin, observational fear, empathy, sex differences, anterior insular cortex, chemogenetics

## Abstract

**Background:** Observational fear, a form of empathic response to others’ distress, exhibits marked sex differences. Oxytocin (OT) is a key modulator of social and emotional behaviors, but its role in observational fear—and how this varies by sex and administration route—remains controversial. **Methods:** We studied behavioral responses in male and female mice during observational fear. We first blocked systemic oxytocin (OT) signaling with a peripheral antagonist. We then tested different routes of OT administration (intranasal, intraperitoneal). Further, we microinjected OT directly into the anterior insular cortex (AIC). Finally, we used a chemogenetics strategy to selectively activate or inhibit OT neurons. **Results:** Male mice exhibited sustained freezing behavior and elevated corticosterone levels in response to observational fear. In contrast, females more quickly resumed baseline activity levels and showed an increased number of interactions. Systemic blockade of oxytocin (OT) signaling selectively reduced fear expression in males. Strikingly, intranasal OT administration elicited heightened fear-related responses in both sexes, whereas intraperitoneal OT administration induced anxiolytic-like effects. Direct OT microinjection into the anterior insular cortex (AIC) produced sex-divergent reductions in fear responses: decreasing freezing duration in males and reducing avoidance behaviors in females. Chemogenetic activation of OTergic neurons replicated these anxiolytic effects, while inhibition had no effect. **Conclusions:** OT bidirectionally regulates observational fear in a sex-, route-, and site-specific manner, challenging the simplistic view of OT as universally prosocial. The AIC is a critical node in empathetic fear circuits. These findings underscore the necessity for precision in targeting the OT system for treating stress-related psychiatric disorders.

## 1. Introduction

Observing others in distress often elicits a personal distress response [1]. Sex differences, potentially driven by natural hormonal variations, may influence the perception of social stressors. Although it is commonly held that women exhibit greater empathy than men in response to others’ adversity, this view remains debated. Furthermore, growing evidence supports the critical role of oxytocin (OT) as a modulator of emotional perception [2]. However, little is known about oxytocin’s sex-specific effects contributing to variability in empathic behavior.

Oxytocin, primarily synthesized in the paraventricular (PVN) and supraoptic nuclei (SON), is critically involved in social behavior, stress regulation, and mental health, as evidenced by both human and animal studies. OT-deficient mice exhibit heightened anxiety-like behavior and an exaggerated corticosterone response to stress, indicating hyperactivity of the HPA axis [3,4]. In humans, acute OT administration combined with social support reduced anxiety and cortisol responses during the Trier Social Stress Test [5]. Nevertheless, OT’s influence on emotion perception shows inconsistency across studies. For instance, intracerebroventricular OT administration enhanced pair-bonding in female prairie voles but not males [6], while intracerebral OT injections benefited male rats but not females, suggesting greater male sensitivity to exogenous OT [7]. Intranasal OT impaired social recognition memory and emotion perception in men but not women [8], and neuroimaging studies reveal OT-induced differences in brain activation patterns between sexes [9,10]. These findings also suggest the existence of brain region-specific sex differences in how OT modulates emotional perception.

The anterior insular cortex (AIC), a hub for interoception, is robustly activated during the observation of distress in others [11,12]. Lesions to the insula impair emotional recognition and empathy [13,14], underscoring its vital role in empathetic processing. Recent research positions OT as a crucial modulator of interoception [15]. The insular cortex receives direct OTergic projections from the PVN [16] and exhibits enriched oxytocin receptor (OTR) binding [17]. Crucially, pharmacological blockade of insular OTRs prevents social affective behaviors in rats, while local OT administration reinstates them [18], highlighting the AIC as a key site for OT’s action on empathy-related circuits. Paradoxically, despite OT’s pro-social reputation, one neuroimaging study found that intranasal OT significantly reduced empathy-related activation in pain neural circuits, particularly within the left insula [19]. This discrepancy in OT’s effects on pain empathy may stem from differences in administration routes (central vs. peripheral). Consequently, understanding how central versus peripheral OT administration modulates social fear and whether these effects are sex-specific is essential.

Therefore, to address the significant gap in understanding OT’s sex-specific effects via different routes and the potential role of the AIC, we employed an observational fear model—a standard paradigm for studying physiological stress-induced fear behavior. This study specifically investigated behavioral differences in observational fear between male and female mice following peripheral or central OT administration. Our findings will provide critical experimental evidence regarding the potential sex-specific application of oxytocin in treating psychosocial stress-related disorders.

## 2. Result

### 2.1. Male and Female Observers Show Different Observational Fear Behaviors

**Figure 1:**
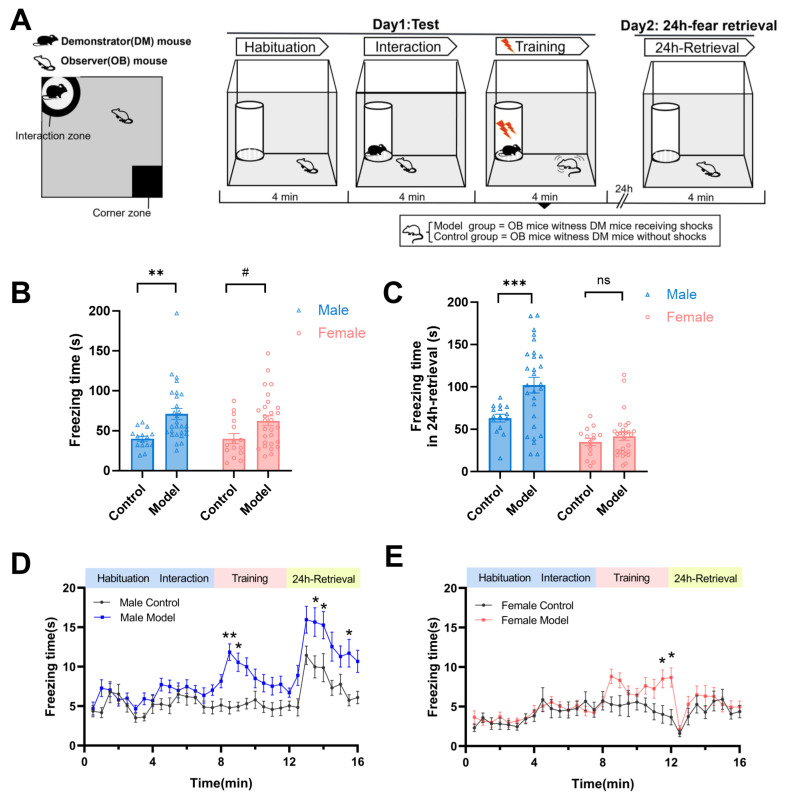
Sex-dependent observational fear behaviors in mice. (**A**) Schematic representation of the observational fear paradigm and behavioral protocol in mice. (**B**) Freezing time during training. Both male and female model mice (observers of shocked demonstrators) showed significantly higher freezing than sex-matched controls (observers of non-shocked demonstrators) (Main effect: F_(1,82)_ = 14.94, for males, ** *p* < 0.01, for females, ^#^ *p* < 0.05). (**C**) Freezing time during 24-h retrieval. Males exhibited amplified freezing (*** *p* < 0.001 vs. control), while females fully recovered to baseline levels. (**D**,**E**) Temporal dynamics of freezing during training trial. Data represent mean ± SEM (control group n = 15; model group n = 28). Statistical significance: * *p* < 0.05, ** *p* < 0.01, two-way ANOVA with post hoc comparison. ns for no significance.

We investigated observational fear behaviors in observer (OB) mice of both sexes, quantifying freezing, active avoidance (corner zone time), and social interaction during Day 1 testing (observational fear training) and 24-h fear retrieval on Day 2 (Figure 1A). Model OB mice observed demonstrators receiving foot shocks, while control OB mice observed non-shocked demonstrators. On observational fear training (Day 1), both male and female model mice exhibited significantly higher freezing times compared to their sex-matched controls during the training stage (*F*_(1,82)_ = 14.94, *p* < 0.001, Figure 1B). However, during the 24 h-fear retrieval, sex-dependent differences emerged: male mice showed significantly amplified freezing time (*p* < 0.001 vs. control group), whereas females exhibited full recovery of freezing behavior (Figure 1C). Temporal analysis further revealed distinct behavioral trajectories in the training stage. Males showed immediate freezing post-initial shocks, whereas females exhibited delayed freezing that reached significance only in later trials (model vs. control, *p* < 0.05, Figure 1D,E).

**Figure 2:**
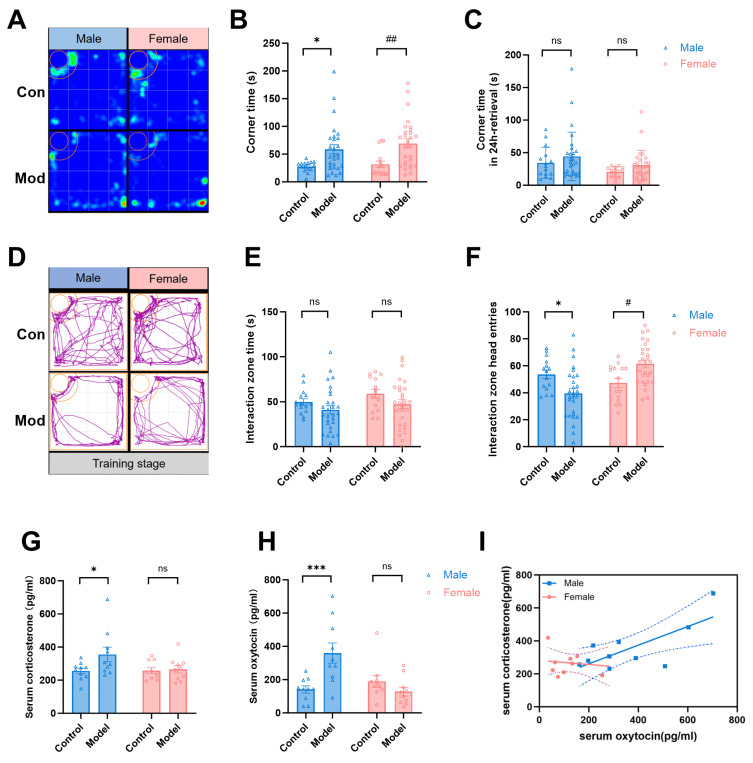
Sex-specific patterns in active avoidance, social behaviors, and hormonal responses during observational fear: (**A**,**B**) Active avoidance (corner zone time) during observational fear training: Both male and female model mice exhibit significantly increased avoidance compared to sex-matched controls (Main effect: F_(1,82)_ = 16.81, * *p* < 0.05, ^##^ *p* < 0.01), indicating acute escape responses. (**C**) Active avoidance during 24-h fear retrieval: No significant differences in corner zone time between model and control groups for either sex (Males: *p* = 0.461; Females: *p* = 0.433), demonstrating transient avoidance limited to the training phase. (**D**,**E**) Social interaction during training: Model groups (both sexes) do not show significant social avoidance versus control. (**F**) Males display reduced entries into the interaction zone (* *p <* 0.05), while females exhibit increased entries (^#^ *p <* 0.05). (**G**) Serum corticosterone (Cort) levels significantly elevated in male model mice versus controls (* *p* < 0.05) but unchanged in females post-training. (**H**) Serum oxytocin (OT) levels: Male model mice have higher concentrations than male controls (*** *p* < 0.001), with no change in females. (**I**) Correlation analysis: Positive correlation between serum OT and Cort in male model mice (r^2^ = 0.4411, *p* < 0.05) but no correlation in females (r^2^ = 0.0217, *p* > 0.05), suggesting sex-divergent neuroendocrine adaptations. ns for no significance.

We next evaluated active avoidance behavior using corner zone time. During the observational fear training stage, both male and female model mice exhibited significantly increased corner zone time compared to sex-matched controls (*F*_(1,82)_ = 16.81, *p* < 0.001, Figure 2A,B), indicating escape behavior upon witnessing distress. However, during 24-h fear retrieval, this avoidance pattern dissipated, and no significant differences in corner zone time were observed between model and control groups in either sex (*F*_(1,82)_ = 2.612, Males: *p* = 0.461; Females: *p* = 0.433, Figure 2C), suggesting transient avoidance responses specific to the acute observation phase.

During the initial two stages of social interaction testing, both male and female observer mice exhibited significant social preferences for familiar partners over empty transparent cylinders (*p* < 0.05; Figure S1). In the training phase, model groups (both sexes) did not display significantly more social avoidance behaviors than control groups (Model vs. Control: *F*_(1,82)_ = 3.867, *p* = 0.0526, Figure 2D,E). Correspondingly, male mice also showed reduced entries into the interaction zone (*p* < 0.05). Interestingly, female mice showed significantly increased entries, with elevated shuttling frequency between zones (*p* < 0.05, Figures S2). These findings further reveal sex-specific behavioral adaptations in social engagement strategies.

We then found a significantly increased serum corticosterone (Cort) in male model mice immediately after the observational fear training (*F*_(1,36)_ = 3.780, Males: *p* < 0.05, Figure 2G). And we also found that male model mice had significantly higher oxytocin (OT) concentrations than male control group (*F*_(1,36)_ = 4.006, Males: *p* < 0.001, Figure 2H). However, these changes were not observed in female mice. Further, correlation analysis showed a positive correlation between serum oxytocin and corticosterone levels in the male model mice (*r*^2^ = 0.4411, *p* < 0.05) but no correlation in the female model mice (*r*^2^ = 0.0217, *p* > 0.05, Figure 2I). Females may maintain homeostasis through behavioral compensation rather than elevated hormonal responses, as evidenced by their heightened exploratory behavior and increased shuttle frequencies during social fear.

### 2.2. Peripheral Administration of Oxytocin Antagonist Reduces Observational Fear

**Figure 3:**
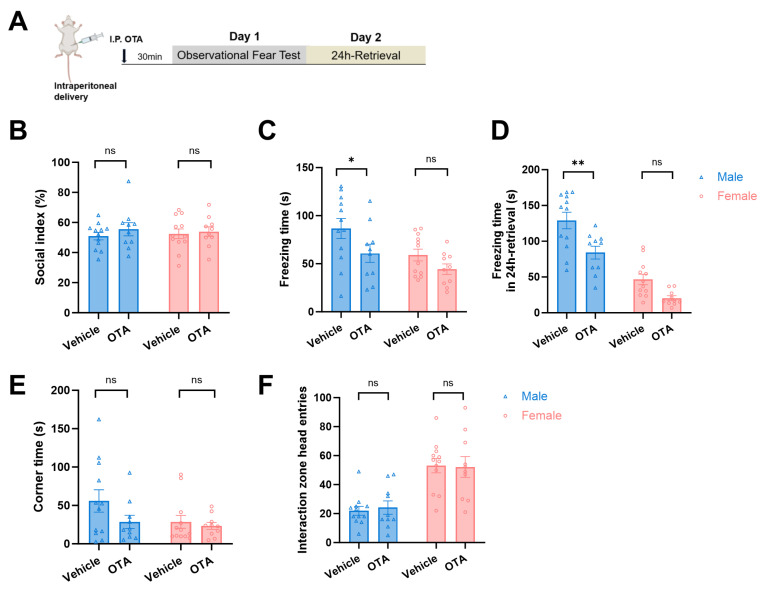
Peripheral oxytocin antagonism reduces observational fear in male mice: (**A**) Experimental timeline: Oxytocin antagonist (OTA, L-368899) or vehicle administered intraperitoneally 30 min before observational fear. (**B**) Social preference index pre-test: No significant differences between OTA and vehicle groups in either sex (*p* > 0.05), confirming intact baseline sociability. (**C**) Freezing during observational fear training: OTA significantly reduces freezing in male mice versus vehicle controls (* *p* < 0.05) with no effect in females. (**D**) Freezing during 24-h retrieval: OTA-treated males exhibit significantly less freezing than vehicle controls (** *p* < 0.01), while females show no significant difference. (**E**) Corner time remained unaltered in both sexes. (**F**) Head entries during social interaction: OTA reverses observational fear-induced alterations in exploratory behavior, normalizing head entries in both sexes versus vehicle (Males: *p* = 0.9376; Females: *p* = 0.9904). ns for no significance.

Considering that elevated oxytocin may be involved in fear regulation, we hypothesized that oxytocin antagonists can alleviate observational fear in male mice. Here, the oxytocin antagonist (OTA) L-368899 was intraperitoneally injected into observer mice 30 min before the observational fear test (Figure 3A). The social preference index was calculated first, and it was shown that male and female mice in each group had similar social preference (*p* > 0.05, Figure 3B). During observational fear testing, OTA significantly reduced freezing in male mice (*F*_(1, 40)_ = 5.993, *p* = 0.0188, Males: *p* < 0.05, Figure 3C). On the 24 h-fear retrieval test, OTA-treated males exhibited significantly less freezing than vehicle controls, whereas females showed no significant difference (Vehicle vs. OTA group, *F*_(1, 40)_ = 16.92, for males: *p* = 0.0014, for females: *p* = 0.0744, Figure 3D). OTA administration did not alter corner time and head entries for both sexes (Vehicle vs. OTA group, *F*_(1, 40)_ = 0.0019, for males: *p* = 0.9376, for females: *p* = 0.9904, Figure 3E,F). These results indicate that peripherally inhibited endogenous oxytocin reduces observational fear in males.

### 2.3. Differential Effects of Oxytocin Administration Routes on Observational Fear

**Figure 4:**
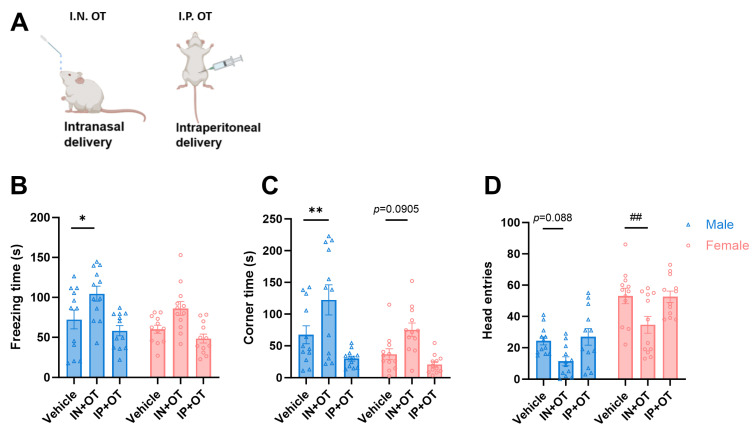
Route-dependent effects of exogenous oxytocin on observational fear behaviors: (**A**) Experimental design: Oxytocin administered via intranasal (IN) or intraperitoneal (IP) prior to observational fear testing. (**B**) Freezing duration in males: IN oxytocin significantly increases freezing versus vehicle controls (* *p* < 0.05). IP oxytocin shows a non-significant trend toward reducing freezing. (**C**) Corner zone time in males: IN oxytocin significantly increases avoidance (** *p* < 0.01) with a concurrent trend toward increased corner time in female mice (*p* = 0.0905). (**D**) Interaction zone entries in females: IN oxytocin significantly reduces exploratory behavior (^##^ *p* < 0.01).

To investigate the effects of exogenous oxytocin (OT) on social fear, we administered OT either intranasally (IN) or intraperitoneally (IP) to observe mice prior to observational fear testing and assessed fear-related behaviors (Figure 4A). The results revealed distinct effects based on the administration route. Intranasal oxytocin significantly increased social fear in male mice, as evidenced by the following: a significant increase in freezing duration (Vehicle vs. IN + OT group, *F*_(2, 66)_ = 13.99, for males: *p* < 0.05, Figure 4B), a significant increase in the time spent in the corner (*F*_(2, 66)_ = 17.04, for males: *p* < 0.01, Figure 4C), and a trend towards decreased entries into the interaction zone. Conversely, intraperitoneal oxytocin administration showed a trend towards reducing fear in male mice, although this effect did not reach statistical significance. In female mice, intranasal oxytocin similarly promoted fear-related behaviors: it increased the time spent in the corner (trend) and significantly reduced the number of entries into the interaction zone (Vehicle vs. IN + OT group, *F*_(2, 66)_ = 9.538, for females: *p* < 0.01, Figure 4D). These findings demonstrate that exogenous OT administration potentiates observational fear behavior in both male and female mice. However, this pro-fear effect is critically dependent on the route of administration, being most pronounced with intranasal delivery. In contrast, intraperitoneal injection exhibited a non-significant trend towards reducing fear in males, suggesting divergent central nervous system effects based on the delivery pathway.

### 2.4. AIC Oxytocinergic Neurons Mediate Sex-Divergent Modulation of Observational Fear Responses

**Figure 5:**
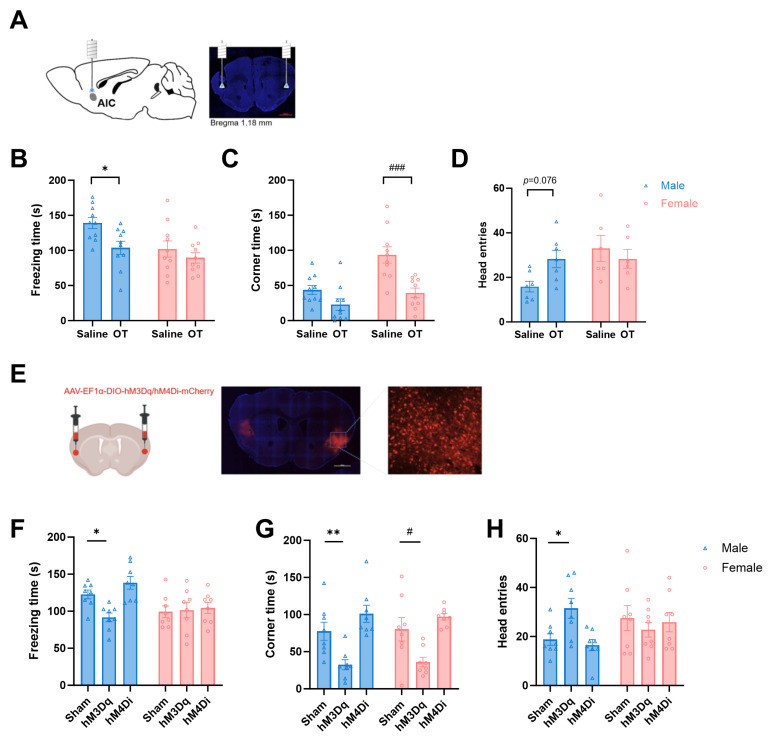
Anterior insular cortex (AIC) oxytocinergic neurons mediate sex-divergent modulation of observational fear: (**A**) Cannula placement verification in AIC (post-hoc immunohistochemistry). (**B**–**D**) Effects of bilateral AIC oxytocin (OT) microinjection: Males: Reduced freezing time versus saline controls (* *p* < 0.05). Females: Significantly decreased corner time (^###^ *p* < 0.001), but no changes in interaction zone entries in either sex. (**E**) Histological confirmation of AAV-EF1α-DIO-hM3Dq-mCherry/hM4Di-mCherry expression in AIC-OT neurons of OT-Cre mice. (**F**–**H**) Chemogenetic activation (hM3Dq) of AIC-OT neurons: Males: Reduced freezing (* *p* < 0.05), decreased corner time (** *p* < 0.01), and increased head entries (* *p* < 0.05). Females: Decreased corner time (^#^ *p* < 0.05), freezing and entries unchanged. Chemogenetic inhibition (hM4Di): No significant behavioral changes in either sex vs. sham controls.

Previous studies on the neural mechanisms of empathetic pain have identified the anterior insular cortex (AIC) as a core region for processing observational pain [20,21]. To investigate whether oxytocin (OT) signaling in the AIC modulates observational fear behavior, we bilaterally microinjected OT into the AIC via implanted cannula 30 min prior to behavioral testing. Cannula placement was verified post hoc by immunohistochemistry (Figure 5A). We found that region-specific OT administration reduced observational fear in both sexes. Male mice exhibited reduced freezing time (Saline vs. OT group, *F*_(1, 36)_ = 6.844, for males: *p* < 0.05, Figure 5B), while female mice significantly reduced the time spent in the corners (Saline vs. OT group, *F*_(1, 36)_ = 19.47, for females: *p* < 0.001, Figure 5C). And there was no significant difference in the number of head entries into the interaction zone (Figure 5D).

To determine if these effects were mediated by AIC oxytocinergic neurons, we utilized OT-Cre mice (Jackson Laboratories, ID 0242343) expressing Cre recombinase downstream of the OT gene stop codon. Following bilateral AIC injection of AAV-EF1α-DIO-hM3Dq-mCherry (activation) or AAV-EF1α-DIO-hM4Di-mCherry (inhibition), viral expression sites were histologically confirmed (Figure 5E). After 3 weeks of recovery, CNO (5 mg/kg, i.p.) was administered 30 min pre-test to chemogenetically manipulate neurons. Chemogenetic activation of oxytocinergic projections to the AIC recapitulated OT effects with notable sex differences: male mice showed significantly reduced freezing time (*F*_(2, 42)_ = 5.105, *p* < 0.05, Figure 5F), decreased corner time (*F*_(2, 42)_ = 20.76, *p* < 0.01, Figure 5G), and increased head entries (*F*_(2, 35)_ = 1.421, *p* < 0.05, Figure 5H). Female mice only show decreased corner time (*p* < 0.05). However, chemogenetically inhibiting the AIC oxytocinergic neurons, there was no significant difference for male and female mice between the Sham and hM4Di groups. In short, AIC oxytocinergic neurons are sufficient but not necessary for observational fear modulation, with males showing broader behavioral sensitivity than females. This highlights sex-specific circuit mechanisms for OT-driven fear regulation.

## 3. Methods

### 3.1. Subjects

Adult male C57BL/6J mice (18–23 g, 8 weeks old) and female C57BL/6J mice (15–20 g, 8 weeks old, n = 43) were acquired from Beijing Sibeifu Animal Company (Animal Licence No. SCXK2019-0010; Beijing, China). The mice of different sexes were separately housed in groups (4 animals/cage) and maintained under the standard conditions (12/12 h light/dark cycle, 22 ± 2 °C, water and food ad libitum). All mice adapted to ambient rearing conditions for 7 days before the experiments. Mice of each sex were randomly assigned to control (n = 15) and model (n = 28) groups. Control group animals were exclusively used for model validation and were humanely euthanized after behavioral testing. Model group animals were first subjected to model validation. Among these, a subset (n = 8) that met predetermined criteria for successful fear acquisition (freezing response threshold >30%) was subsequently allocated to chemogenetic intervention groups (e.g., hM3Dq, hM4Di). This approach ensured continuity in baseline behavioral profiles while minimizing inter-cohort variability.

### 3.2. Observational Fear Paradigm

The experimental chamber is a closed device 40 cm × 40 cm × 45 cm (long × wide × high). The bottom is a waterproof whiteboard, and in the upper left corner is a transparent openwork cylinder with a diameter of 10 cm and a height of 8 cm for animals to interact with and sniff. A conductive metal grid beneath the whiteboard delivers plantar shocks to mice in a cylinder, as previously described [22]. A digital camera was mounted on top of the device to record animal behavior in various areas of the device. On day 1, each observer (OB) mouse was allowed for 4 min of habituation in the apparatus. In the social interaction phase, a same-sex partner demonstrator (DM) mouse was placed in the cylinder, allowing the mice to interact and sniff for another 4 min. During the training phase, the OB mouse witnessed the DM mouse receiving foot shocks (each 1.0 mA, 2 s long) over 4 min and being able to move freely within the chamber. The inter-shock interval was 10 s. Control OB mice undergo the same procedure but witness a DM mouse without electric foot shock. After training, the animals were returned to their home cages. On day 2, the OB mouse was returned to the chamber and recorded for 4 min of free activity during the retrieval. We scored the OB mouse’s freezing, avoidance, and social interaction behaviors over two days of experiments (Figure 1A). Distance ratio was calculated as interaction zone distance/total distance, and the social index = Time with DM mouse (interaction)/[Time with empty cup (habituation) + Time with DM mouse (interaction)].

### 3.3. Drug Application

Oxytocin (MCE Cat. #HY-17571A) was dissolved in saline in aliquots at −80 °C. The oxytocin antagonist (OTA, L-368,899 hydrochloride, MCE Cat. #HY-108677) was dissolved in 1 mg/mL saline and frozen at −20 °C in aliquots. Mice were given oxytocin (20 μg/kg) or saline of 20 μL per nostril intranasally. Mice were given the OXTA L-368,899 hydrochloride (10 mg/kg, I.P.) for the systemic oxytocin antagonist. Clozapine N-oxide (MCE Cat. #HY-17366) was dissolved in 0.5 mg/mL saline and frozen at −20 °C in aliquots. For the systemic CNO, mice were given the CNO (5 mg/kg, I.P.) to selectively activate or inhibit oxytocinergic neurons in the anterior insular cortex (AIC) of Oxt-cre mice, allowing researchers to investigate their role in observational fear behavior. Intranasal oxytocin (OXT) administration: Synthetic oxytocin (OXT) was dissolved in sterile saline and administered intranasally at a dose of 20 µg/kg. A total volume of 4 µL was delivered bilaterally (2 µL per nostril) over a period of 2 min using a micropipette fitted with a gel-loading tip to ensure precise and non-invasive application. Intraperitoneal (i.p.) administration: All intraperitoneal injections, including saline vehicle controls, were administered at a standardized volume of 5 mL/kg of body weight. Bilateral microinjection into the anterior insular cortex (AIC): For localized drug delivery, OXT (or its antagonist) was microinjected bilaterally into the AIC at a concentration of 100 µM. A volume of 0.5 µL per side was infused into each hemisphere over 2 min (0.25 µL/min) using a microprocessor-controlled microinfusion pump. To minimize reflux and allow for complete diffusion of the solution, the injection cannula was left in place for an additional 2 min following each infusion. All animals receiving oxytocin (via intranasal, intraperitoneal, or central routes) or its antagonist (L-368,899, i.p.) were naïve mice that had not previously undergone any behavioral testing, ensuring no confounding effects from prior experimental exposure.

### 3.4. ELISA Kit to Detect Mouse Serum

Prior to ELISA, serum samples underwent acid–ethanol extraction according to the manufacturer’s instructions to improve detection specificity. All assays were run in duplicate, and values were interpolated from standard curves using four-parameter logistic regression. Assay performance was validated based on internal quality controls provided by the kit. After the completion of the behavioral experiment, the mouse eyeball blood was taken from mice on the first day of testing. Then, the blood was stored overnight in a 4 °C freezer, centrifuged at 3000× *g* at 4 °C for 15 min, the supernatant was aspirated, aliquoted into 200 μL EP tubes, and stored in a −80 °C refrigerator for later use. Follow the Mouse CORT (Corticosterone) ELISA Kit (Elabscience, E-OSEL-M0001, Wuhan, China) and OT (Oxytocin) ELISA Kit (Elabscience, E-EL-0029c, Wuhan, China) instructions exactly.

### 3.5. Anterior Insular Cortex Cannula Placement and Microinjection

Cannulation and microinjection methods were performed as previously described [23]. Briefly, mice were anesthetized by 0.9% nembutal (60 mg/kg) intraperitoneally. The head of the mouse was fixed into a stereotaxic frame, and an incision was made over the skull to expose the surface. Guide cannulas (27 G, RWD, China) were inserted bilaterally into the AIC (from bregma: AP: 1.75 mm, ML: ±2.75 mm, DV: −4.0 mm from skull surface) and fixed in place with acrylic cement and stainless-steel screws. Microinjection was conducted using a motorized syringe pump (Razel Scientific Instruments, Stamford, CT, USA) and a microsyringe (Hamilton, Reno, NV, USA). Oxytocin (100 μM) or saline alone as the vehicle was delivered bilaterally into the AIC (0.5 μL/side over 8 min). The volume delivered was confirmed by observing the downward movement of the meniscus in calibrated polyethylene (PE6121) tubing. The injector was kept in place for 2 min to help prevent any solution from flowing back up the guide. After microinjection, the mice were returned to their cage for 30 min prior to the behavioral observations.

### 3.6. Chemogenetics

Mice were anesthetized with sodium pentobarbital (60 mg/kg) intraperitoneally. Virus AAV-EF1α-DIO-hM3Dq-mCherry (AAV2/8, 1.49 × 10^13^ genomic copies per mL, OBiO Technology, Shanghai, China) or AAV-EF1α-DIO-hM4Di-mCherry (AAV2/8, 9.98 × 10^12^ genomic copies per ml, OBiO Technology, Shanghai, China) was injected into the AIC (from bregma: AP: 1.75 mm, ML: ±2.75 mm, DV: −4.0 mm from skull surface, 200 nL/side, 50 nL/min) in adult Oxt-cre mice, respectively. These viral vectors were used to enable chemogenetic activation or inhibition, respectively, of oxytocinergic neurons in the AIC. The injector was kept in place for 2 min to help prevent any solution from flowing back up the guide. Two or three weeks after injection, mice can undergo behavioral experiments. These viral vectors are typically used for precise manipulation of neuronal activity, such as activating or inhibiting specific types of neurons with functional consequences.

### 3.7. Immunohistochemistry

To detect neurons activated in mice witnessing cagemates’ pain, we examined c-Fos expression. Mice were sacrificed 90 min after the OFL test (to coincide with the peak of c-Fos expression, 90–120 min post-stimulus) by deep anesthesia with sodium pentobarbital (60 mg/kg, i.p.) and transcardially perfused with heparinized saline followed by 4% paraformaldehyde (PFA). Brains were removed, post-fixed in 4% PFA at 4 °C overnight, and cryoprotected in 30% sucrose/PBS until sinking. Coronal sections (40 µm) were cut on a freezing microtome (CM 1950, Leica, Wetzlar, Germany) and stored in PBS at 4 °C. For immunostaining, free-floating sections were incubated overnight at 4 °C with rabbit anti-c-Fos primary antibody (1:200; Cat#2250s, CST, Danvers, MA 01923, USA), followed by 1 h at room temperature with Alexa Fluor 488 goat anti-rabbit secondary antibody (1:1000; Cat#A11034, Invitrogen, Waltham, MA 02451, USA).

### 3.8. Statistical Analysis

All data are represented as mean ± SEM (GraphPad Prism 9.3.0). Statistical comparisons between the two groups were performed using the unpaired *t*-test. Statistical analyses between multiple groups were performed using one-way ANOVA or two-way ANOVA followed by Sidak’s multiple comparison tests when the main effects of sex were observed to be significant, to identify significant differences. In all cases, *p* < 0.05 was considered statistically significant. All behavioral scoring and data analyses were performed by experimenters blinded to group allocation.

## 4. Discussion

In the present study, we systematically investigated sex-specific differences in observational fear behavior and the modulatory role of oxytocin (OT) via different administration routes, with a particular focus on the involvement of anterior insular cortex (AIC) OTergic signaling. Our findings reveal several key insights: (1) male and female mice exhibit distinct behavioral and neuroendocrine responses in observational fear, characterized by sustained freezing in males and rapid behavioral recovery in females; (2) peripheral blockade of OT signaling attenuates observational fear selectively in males; (3) intranasal OT exacerbates fear-related behaviors in both sexes, whereas central OT delivery into the AIC reduces fear expression; and (4) chemogenetic activation of oxytocinergic projections to the AIC mimics the anxiolytic effect of intra-AIC OT infusion, with pronounced sex-divergent outcomes. Together, these results highlight a critical, route- and sex-dependent role for oxytocin in social fear modulation, positioning the AIC as a pivotal node in the neural circuitry underlying empathetic fear responses.

Our observation of sexually dimorphic fear expression aligns with growing evidence that males and females employ different coping strategies in response to social stressors [24]. Specifically, male observer mice displayed prolonged freezing both during and after witnessing conspecific distress—a hallmark of persistent fear memory—while females showed transient freezing that normalized within 24 h. This divergence suggests that females may utilize more adaptive regulatory mechanisms to restore emotional homeostasis following vicarious stress exposure. Notably, female mice exhibited increased exploratory shuttling and interaction zone entries during observational fear, indicating enhanced behavioral flexibility. These findings resonate with clinical observations that women tend to engage more actively in social processing and emotion regulation, while men may be predisposed to passive defensive reaction [25].

The contrasting effects of peripheral versus central OT administration underscore the importance of delivery route in determining functional outcomes. Intranasal OT, widely used in translational studies for its presumed ability to enhance central OT levels, unexpectedly intensified observational fear in both sexes. This paradoxical pro-fear effect challenges the prevailing notion of OT as a universally prosocial and anxiolytic molecule, suggesting that non-physiological surges of OT may disrupt finely tuned neural circuits involved in social cognition [26]. In contrast, intraperitoneal OT showed a trend toward fear reduction, possibly due to slower pharmacokinetics and more stable systemic exposure. Intranasal OT is thought to rapidly access the central nervous system via the olfactory pathway or the trigeminal nerve. This results in a non-physiological high-concentration surge of OT in local brain regions over a short time frame. Such a sudden and intense increase may over-activate or even desensitize receptors, ultimately disrupting the normal social and emotional regulation circuits. In contrast, intraperitoneal OT is absorbed at a relatively slow pace. Once it enters the circulatory system, it distributes more evenly. This leads to a gradual and sustained increase in OT levels that are close to physiological norms, mimicking the endogenous release of OT. As a result, it can play a regulatory role rather than causing interference in the body’s normal functions. Importantly, direct microinjection of OT into the AIC produced clear anxiolytic effects—reducing freezing in males and avoidance in females—supporting the hypothesis that localized OT release in this region promotes resilience to social fear.

The AIC emerges as a central hub integrating interoceptive awareness, emotional salience, and empathy-related processing [27,28]. Its dense innervation by OTergic fibers from the paraventricular nucleus (PVN) and high expression of oxytocin receptors (OTRs) position it as a prime target for OT-mediated modulation of social emotion [29,30]. Our chemogenetic experiments confirm this functional link: selective activation of OT-expressing neurons projecting to the AIC recapitulated the fear-reducing effects of local OT infusion, particularly in males who exhibited reduced freezing and avoidance. This sex bias may reflect intrinsic differences in OT receptor density, synaptic connectivity, or hormonal milieu—factors known to influence OT system sensitivity [16,17]. Indeed, estrogen has been shown to upregulate OTR expression in limbic regions, potentially enabling faster desensitization or feedback inhibition in females [31].

Furthermore, our finding that systemic OT antagonism alleviates observational fear exclusively in males implies a tonic facilitatory role of endogenous OT in male social fear circuits—an effect not mirrored in females. This contrasts with traditional views of OT as uniformly prosocial and highlights the necessity of considering sex as a biological variable in neuropsychopharmacology. Given that OT-synthesizing somata are primarily located outside the AIC, the observed effects likely reflect modulation of incoming OTergic axon terminals from upstream nuclei such as the PVN, though direct immunohistochemical confirmation of viral expression in OT+ fibers within the AIC was not performed. The lack of hormonal response (i.e., corticosterone elevation) in female observers, despite comparable initial distress perception, suggests that females may rely more heavily on behavioral compensation rather than neuroendocrine activation to manage vicarious stress. This adaptive phenotype could contribute to lower vulnerability to certain trauma-related disorders, though it may also predispose to internalizing conditions under chronic stress [32,33].

## 5. Conclusions

Nonetheless, our work provides compelling experimental evidence for sex-specific and route-dependent effects of oxytocin in modulating empathic fear. These findings caution against the indiscriminate use of intranasal OT in clinical settings, especially without regard to patient sex or underlying neural circuit state. Instead, they advocate for targeted interventions—such as region-specific neuromodulation or personalized dosing regimens—that respect the nuanced biology of the oxytocin system. Ultimately, understanding how OT shapes social emotional responses in a context- and sex-dependent manner may pave the way for more effective treatments for psychosocial stress-related disorders.

## 6. Limitations of This Study

Despite its strengths, this study has limitations. First, all experiments were conducted in naïve adult C57BL/6J mice under controlled laboratory conditions, which may not fully capture the complexity of naturalistic social interactions. Second, while we focused on the AIC, other regions such as the anterior cingulate cortex, amygdala, and bed nucleus of the stria terminalis are also implicated in observational fear and likely interact with OTergic pathways. Third, while we utilized Oxt-Cre mice for targeted manipulation, we did not provide histological evidence confirming colocalization of mCherry-labeled fibers with oxytocin immunoreactivity within the AIC. Thus, the precise anatomical origin and neurotransmitter phenotype of the manipulated projections require further validation via retrograde tracing or dual-immunofluorescence studies. Fourth, changes in serum OT levels should be interpreted with caution, as peripheral concentrations may not reliably reflect central OT dynamics or neuronal release. We did not measure central OT concentrations directly, relying instead on behavioral and pharmacological proxies. Future studies employing microdialysis or fiber photometry could elucidate real-time dynamics of OT release during social threat observation. Fifth, our use of intraperitoneal oxytocin administration, while effective in modulating social investigation and fear acquisition, exhibits slower and less specific central nervous system kinetics compared to intranasal delivery. Future studies utilizing intranasal administration (15–20 min prior) could provide more targeted central engagement and potentially reveal stronger or more rapid effects on observational fear dynamics.

## Data Availability

The original contributions presented in this study are included in the article/Supplementary Material. Further inquiries can be directed to the corresponding author(s).

## Supplementary Materials

The following supporting information can be downloaded at https://www.mdpi.com/article/10.3390/ph19030350/s1, Figure S1: Social preference index in male and female groups.; Figure S2: (A) Social index in male and female groups. (B) Total distance travelled during training stage in male and female groups.
