# Supplementary material for: Divergent Effects of Peripheral vs. Central Oxytocin Administration on Observational Fear Behavior in Male and Female Mice
Source: Pharmaceuticals (Basel). 2026 Feb 24;19(3):350. doi: 10.3390/ph19030350 (PMC13029319; doi:10.3390/ph19030350)
Supplement: Supplementary file 1 [file pharmaceuticals-19-00350-s001.zip › pharmaceuticals-4070517-supplementary.pdf]

Supplementary Materials: Divergent Effects of Peripheral vs. Central Oxytocin Administration on Observational Fear Behavior in Male and Female Mice

Yuan Fu, Shufang Feng, Wenlong Shi, Yu Qin, Tianyao Shi and Wenxia Zhou

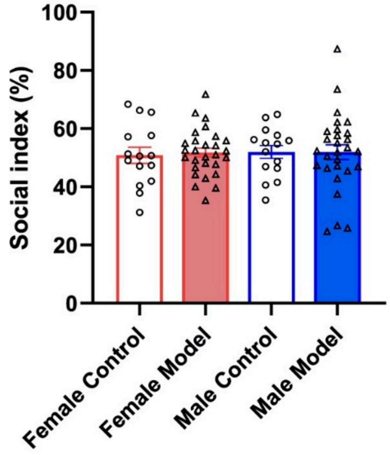

Figure S1. Social preference index in male and female groups. The social index is calculated as: Social index = Time with DM mouse (interaction) / [Time with empty cup (habituation) + Time with DM mouse (interaction)]. Data represent mean ± SEM (control group n=15; model group n=28).

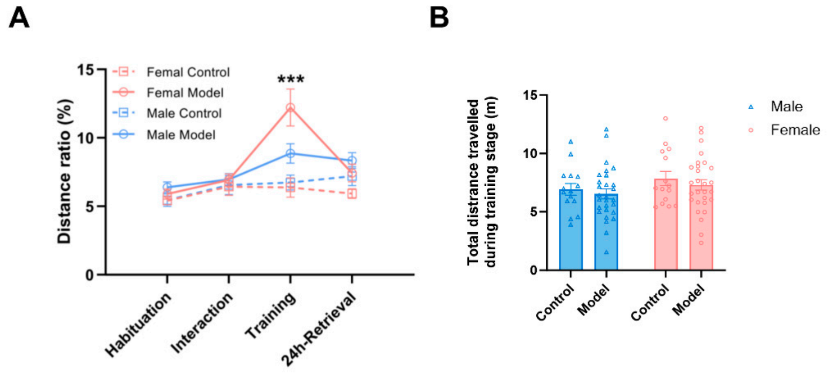

Figure S2. (**A**) Social index in male and female groups. (**B**) Total distance travelled during training stage in male and female groups. Data represent mean ± SEM (control group n=15; model group n=28).
